# Effect of Substitution of Rice Flour with Quinoa Flour on the Chemical-Physical, Nutritional, Volatile and Sensory Parameters of Gluten-Free Ladyfinger Biscuits

**DOI:** 10.3390/foods9060808

**Published:** 2020-06-19

**Authors:** Michela Cannas, Simone Pulina, Paola Conte, Alessandra Del Caro, Pietro Paolo Urgeghe, Antonio Piga, Costantino Fadda

**Affiliations:** Dipartimento di Agraria, Università degli Studi di Sassari, Viale Italia 39/A, 07100 Sassari, Italy; mcannas@uniss.it (M.C.); simopulina@hotmail.it (S.P.); pconte@uniss.it (P.C.); delcaro@uniss.it (A.D.C.); paolou@uniss.it (P.P.U.); cfadda@uniss.it (C.F.)

**Keywords:** volatile compounds, gluten-free biscuits, nutritional value, polyphenols, quinoa flour

## Abstract

The present study investigates the effect of partial or total substitution of rice flour (RF) with quinoa flour (QF) (at 25%, 50%, 75% and 100%) on the chemical-physical, nutritional, and sensory characteristics, as well as the volatile compounds, of ladyfinger biscuits. All quinoa-based formulations positively affected the crust colour, endowing it with lower ‘lightness’ and higher ‘redness’ values, giving the biscuits a more appealing crust colour. Biscuits with higher percentages of QF also had better structure, as they were softer. The substitution of RF with QF significantly improved the nutritional profile of the biscuits, as a result of the increase in protein, lipid, ash, total soluble (SP) and insoluble polyphenol (IP), flavonoid, and antioxidant activity levels, which increased linearly with the substitution rate. Quinoa supplementation led to an increase in volatile compounds that were nearly always characterised by positive olfactory attributes. Sensory analysis revealed that the maximal substitution rate of QF able to maintain an adequate consumer acceptability rating is probably 50%, as higher percentages impaired acceptability due to the presence of herbaceous and bitter tastes, even if the consumers also rated these samples as healthier and softer to touch.

## 1. Introduction

A strict gluten-free (GF) diet is followed by many people who do not suffer from celiac disease nowadays. The exclusion of gluten is considered, by many, to be a healthy habit or a way to prevent the onset of celiac disease [1]. Thus, the food industry is continuously increasing the number of new cereal-based GF foods to offer consumers, whilst continuing to face the well-known technological, nutritional, and sensory problems characteristic of these products [2,3,4,5].

Of the different types of GF bakery foods, biscuits are of great importance from a commercial standpoint [6] due to their ease of use, adequate nutritional content, the wide selection of biscuit types available, their long shelf-life, and their relatively low selling price [7]. Moreover, fewer technological problems are encountered in biscuit production compared with bread or pasta production, as the build-up of gluten is not of fundamental importance, and thus the replacement of gluten-containing flours with GF counterparts is simpler [8]. Nonetheless, at least two major problems hamper the production of GF bakery products: inadequate sensorial acceptability; and an unbalanced nutritional profile resulting from the lower content of several important nutrients, such as minerals (iron, zinc, magnesium and calcium), dietary fibre, and vitamins (folate and B12) [3,4,5]. Moreover, GF baked foods often have a lower resistant starch content, along with a higher glycaemic index, than their gluten-containing equivalents [1,9], thus, they can represent a problem for people suffering from common metabolic disorders, such as obesity and diabetes.

In a recent review, the Di Cairano et al. [10] provided an in-depth overview of conventional versus non-conventional flours used in the production of GF biscuits. Rice, millet, teff and oat are the most studied GF cereals [11,12,13,14]. Other authors have also reported on the use of hemp flour [15] and pseudo cereals, such as amaranth, various beans, buckwheat, and quinoa [16,17,18,19].

Of the above-cited GF flours, special importance is being attributed to quinoa (*Chenopodium quinoa, Wild*)—a crop native to the Andean region, where it has been cultivated for circa 7000 years. Although quinoa is still considered a minor crop, mostly connected to north western South America, in the last few decades its cultivation has spread to more than 95 countries across Africa, Asia, and Europe, including Italy [20]. In particular, the cultivation of quinoa is currently being tested in Sardinia, a major Mediterranean island, with the aim of diversifying agricultural production by sowing alternative crops that are oriented towards new consumption patterns, in comparison to traditional patterns. The rekindled interest in this under-utilised plant species is primarily related to its resistance to abiotic stress, as well as to its very important nutritional profile [20]. Quinoa flour (QF), in fact, has been considered a super food due to its higher protein content and more balanced amino acid composition, with respect to cereal flours. The protein, carbohydrate, fat, and fibre content of QF peak at approximately 16.4%, 75.82%, 12.4% and 3.38%, respectively [21]. It is also considered a rich source of vitamins (riboflavin, tocopherol, and ascorbic acid), minerals (calcium, magnesium, iron, potassium, and zinc), and antioxidant compounds [2,22]. Moreover, since quinoa flour is naturally GF, it may also be used as a healthy and nutrient-dense alternative ingredient in the development of functional GF products.

Very few papers have been published on the use of quinoa derivatives for biscuit or cookie manufacturing, especially in comparison with the number of studies carried out on the use of QF in bread. In addition, to the best of our knowledge, most of the existing studies involved the substitution of wheat flour with QF, but only a small proportion refer to GF biscuits. Wang et al. [23] found that substitution of wheat flour with QF at a rate of 60% resulted in a dark colour, greater hardness, and reduced volume, cohesiveness and chewiness of the resulting cookies. On the other hand, an improvement in the nutritional and sensory properties was found in biscuits made by substituting wheat flour with QF at a maximum rate of 50% [19]. Similar results were obtained by Jan et al. [21], who used a surface response methodology to find the optimized values of fat and sugar, as well a baking temperature and baking time, for gluten free quinoa cookies. With reference to GF biscuits, Brito et al. [24] showed that both QF and quinoa flakes mixed with maize starch improved the hardness of GF biscuits and their nutritional profiles, whereas the addition of flakes alone only had a positive effect on the final volume. Thus, considering the elevated number of biscuit types and related different formulations, further studies are to be expected.

In the present study, the type of biscuit chosen was the ladyfinger, which dates back to the 15th century to the court of the Duchy of Savoy, and for this reason it is called “savoiardo” in Italian. Ladyfingers are a low density, dry, egg-based, sweet sponge biscuit made in an oblong shape with rounded ends. The batter is composed of two phases: the continuous one is the non-aerated batter, while air acts as the dispersed phase. It has widespread use, not only as a ready-to-eat biscuit, but also as the main ingredient in several dessert recipes, including charlottes and the Italian tiramisu. Furthermore, this type of biscuit, being included (with the name of “biscotto di Fonni”) in the list of the Traditional Agri-Food Products (PAT) officially approved by the Ministry of Agricultural, Food and Forestry Policies of Italy, has also strong roots in the Sardinian food tradition. In this context, the aim of the present research is to evaluate the effect of the substitution of rice flour (RF) with QF at increasing levels (up to 100%) on the chemical-physical, nutritional, and sensory characteristics of ladyfinger biscuits, and to correlate the percentages of substitution used with consumer acceptability, which strongly affects the marketability of products.

## 2. Materials and Methods

### 2.1. Batter Formulation

All ingredients, with the exception of QF, were purchased from a local supermarket. A control (Ctrl) and four different quinoa-based (Q) formulations were prepared. The Ctrl formulation was made up of 55% (w/w on flour basis) fresh eggs (Azienda Avicola Monte Acuto di Diego Pinna e C. Snc, Ozieri, Italy), 23% (w/w) RF (Chimab, Campodarsego, Italy), and 22% sucrose. The compositions of QF and RF are reported in Table 1. Whole stone milled QF (Quinoa Marche, Jesi, Italy) was used in the Q formulations, substituting RF at a rate of 25 (Q25), 50 (Q50), 75 (Q75) and 100% (Q100). All ingredients were stored at room temperature, except the eggs that were refrigerated at 4 °C until the time of use.

### 2.2. Batter and Biscuit Preparation

A two-step process was followed to produce the end batter. In the first step, the egg yolks were separated from the whites and combined with the other ingredients with minimum air incorporation. The yolks were first added to 70% of the sugar and mixed at high speed (168 rpm) for 5 min with a KitchenAid Professional mixer (Model 5KSM7990, Whirlpool, MI, USA) equipped with a stainless-steel wire whip. After that, flour was added and mixed with the same machine at 72 rpm for the time needed to combine all the ingredients; the speed was then increased to 136 rpm for 3 min. The egg whites were whipped with the remaining sugar using the same equipment for 2.5 min at maximum speed (200 rpm). In the second step, the two batters were manually combined using slow circular movements of a wooden spoon, from the bottom upwards to allow the greatest air incorporation.

The batter was piped onto a stainless-steel baking pan into the classic ladyfinger biscuit form using a pastry bag equipped with a 12-mm circular opening. The dough was then evenly dusted with sucrose (1.5 g per biscuit) to speed up crust development. The biscuits were baked in an electric oven (Europa, Malo, VI, Italy) for 10 min at 170 °C.

### 2.3. Flour and Biscuit Chemical-Physical and Nutritional Analysis

The flours and biscuits were analysed for moisture, ashes, lipids, total and soluble polyphenols, protein, total carbohydrates, total flavonoids, and antioxidant activity. The water activity (aw) of the cooked biscuits was also determined. Analyses were done in triplicate and the data were expressed as g per 100 g of dry matter, unless otherwise indicated.

#### 2.3.1. Chemical-Physical Analysis

Moisture was determined following the AACC method 44-15.02 [25], ashes by incineration in a muffle furnace at 600 °C following the ICC 104-1 method [26], and lipids by Soxhlet extraction following the ICC 136 method [27]. The aw was measured on homogenized samples with an electronic hygrometer (model Aw-Win, Rotronic, equipped with a Karl-Fast probe, Rotronic, Bassersdorf, Switzerland), previously calibrated in the range of 0.1–0.95 with solutions of LiCl of known activity.

#### 2.3.2. Nutritional Analysis

Total (TP) and soluble polyphenols (SP) were measured from both biscuit and flour samples according to the method reported by Carciochi and Dimitrov [28], using a spectrophotometer (mod. 8453, Hewlett–Packard, Palo Alto, CA, USA) set at 760 nm. Briefly, for the determination of the total phenolic compounds, an aliquot of 3 g of partly dried and ground biscuits (or 2 g of flour) was extracted in the dark under constant agitation (room temperature for 1 h) using 20 mL of a solution of ethanol:water (80:20, *v*/*v*) acidified with hydrochloric acid (1%) until a pH of 1.5 was reached. The obtained extracts were filtered through cellulose acetate syringe filters with a pore size of 0.45 μm (LLG Syringe Filter CA, Carlo Erba, Milano, Italy) and stored at a low temperature (4 °C) before reading. The soluble polyphenol fraction was determined under the same conditions, but using a solution of ethanol:water (80:20, *v*/*v*) at a pH of 6.9. The IP were obtained by subtracting SP from TP [28]. Calibration curves were made using gallic acid, and the results are expressed as mg of gallic acid equivalent (GAE) per 100 g of dry matter.

The nitrogen content was determined using a CHN analyser (CHN 628, LECO, St. Joseph, MI, USA). A sample weighing exactly 80 mg was placed inside an aluminium capsule that was accurately folded, then placed into a foil pan and inserted inside the instrument’s sample holder. The combustion temperature was set at 1050 °C. Before analysis, the instrument was calibrated against certified standards. To obtain the protein content, the results were multiplied by 5.95. Total carbohydrates (TC) were obtained by subtracting the moisture, ash, lipid, and protein contents from 100.

Total flavonoids (TF) were determined using the aluminium chloride (AlCl_3_) colorimetric method that forms stable complexes with flavonoids, thus avoiding other polyphenols being detected [29]. Briefly, a 3 g sample was partly dried and finely ground, then extracted using 10 mL of an ethanol:water solution (30:70, v/v). Two mL of the extract solution was then diluted with 2 mL water, and 0.3 mL 5% sodium nitrite solution was immediately added. Six minutes later, 0.6 mL 10% ALCl_3_ solution was added; this solution was left to react for 5 min before adding 2 mL 1 N sodium hydroxide solution. The sample absorbance was immediately read in a spectrophotometer (mod. 8453, Hewlett–Packard, Palo Alto, CA, USA) at 510 nm against a suitable blank, and the values were calculated with the use of a previously calculated calibration curve and expressed as mg of catechin equivalent (CE) per 100 g of dry matter.

Antioxidant activity (AA) was determined using the 2,2-diphenyl-1-picrylhydrazyl (DPPH) stable radical [30], according to Conte et al. [31]. A 3 g biscuit sample or 2 g of flour were partly dried and finely ground before being extracted under constant stirring with 20 mL (10 mL for flour) of a methanol:water (50:50 v/v) solution acidified with 1 M hydrochloric acid, up to a pH of 2. Obtained samples were centrifuged for 10 min at 2500× *g* and the supernatant was saved. The residual solid was once again extracted using the above cited procedure, using 20 mL (10 mL for QF) of an acetone:water (70:30 v/v) solution. The two extracts were combined and made up to 25 mL (50 mL for QF) with methanol. A 0.3 mL sample was made to react with 2.7 mL of a 6 × 10^−5^ M solution of DPPH· for 1 h at 515 nm and 22 °C to obtain a decrease in absorbance by the radical DPPH. Absorbance readings were taken at minute 1, 5, and then after every 5 min (mod. 8453, Hewlett–Packard, Palo Alto, CA, USA). The scavenging effect was calculated as a percentage of the spectrophotometric decrease in absorbance using the following formula:

% Inhibiton = 100 × (A_0_ − A_60_) × A_0_^−1^ (1)

where *A*_0_ is the absorbance value at time 0, and *A*_60_ the absorbance after 60 min.

### 2.4. Colour and Texture Analysis of Biscuits

#### 2.4.1. Colour

The surface colour of the biscuits was determined using a tristimulus colorimeter (DP-301, Konica Minolta Sensing, Osaka, Japan) fitted with a measuring head CR-300 (Konica Minolta Sensing, Osaka, Japan), using a D65 illuminant and a CIE 10° standard observer angle and calibrated against a white tile supplied with the instrument. L*(lightness), a*(redness) and b*(yellowness) were acquired. Three readings were taken, two in the distal part and one in the centre of the biscuit. For each sample, 10 biscuits were considered.

#### 2.4.2. Texture Analysis

Biscuit texture was evaluated using a texture analyser (TA-XT2 Texture Analyser, Stable Microsystems, Surrey, UK) fitted with a 30 kg load cell. Texture Exponent Software TEE32, version 6.1.10.0 (Stable Micro System, Surrey, UK), was used for data processing. A puncture test was performed on the upper surface top part of at least four biscuits, 24 h after baking.

The test was carried out on the centre of the top of the biscuit using a 3 mm diameter cylinder probe (SMS P/3). The biscuit was placed on the contact plate and was punctured to a depth of 10 mm at a speed of 1 mm/s. Three parameters were considered: the maximum force (N) reached during puncturing; the gradient of the curve up to the first major peak (N/s); and the area under the curve.

### 2.5. Biscuit Volatile Compound Analysis

The determination of volatile compounds was carried out on freshly prepared biscuits 2 h after baking by headspace solid-phase microextraction and gas chromatography/mass spectrometry (GC/MS), as reported by Conte et al. (2020). The identification of analytes was performed both by comparison of retention times and spectra with those of pure standards, when available, and by matching the MS spectra and the experimental linear retention indexes with those reported in the literature and in the libraries (NIST/EPA/NIH 2008; HP1607 from Agilent technologies).

### 2.6. Sensory Analysis

CATA (check-all-that-apply) is a versatile multiple-choice questionnaire in which a list of terms is presented to consumers, who are then asked to select all the terms they consider appropriate for the examined products. This technique has been extensively used in marketing research [32], and has recently been applied in the field of sensory and consumer science to gain information about consumer perceptions about food products [33]. One of the main difficulties in applying this test is the actual development of the CATA terms. The CATA terms can have different meanings: they can have a hedonic connotation, or be related to usage occasions or emotions [34,35]. The terms used in this CATA test were selected following both an analysis of the literature on biscuits, quinoa, and GF products [36,37,38,39], and by performing preliminary tests in the laboratory. In order to better optimize the product development a penalty analysis, which is based on the responses of the consumers to CATA questions for a set of samples and their ideal product, was applied [40]. The aim of the penalty analysis is to identify the drivers of liking, and suggest directions for product reformulation [41].

### 2.7. CATA Test

A total of 103 participants (51% men and 49% women), aged between 17 and 65 years, were recruited on the basis of their knowledge and consumption of this kind of biscuit. Biscuits with different percentages of QF (0%, 25%, 50%, 75%, 100%) were presented at room temperature one at a time to the consumers, in plastic containers labelled with a three-digit code. The samples were presented in a randomized and balanced order, and water was provided between samples. The order of presentation was individually randomized for each participant to avoid carry-over effects [42]; moreover, the order of presentation of CATA terms was balanced between and among the participants, as suggested by Ares et al. [43]. First, the consumers were asked to score their overall liking of the product using a 9-point hedonic scale, where 1 was “dislike very much” and 9 was “like very much” [44]. Next, they completed the CATA test by choosing the terms they considered most appropriate to describe the samples. Finally, the consumers were asked to complete the CATA test describing their ideal biscuit. The sensory terms used were the following: sweet, bitter, herbaceous, soft to touch, soft in mouth, crunchy, dry in mouth, nice colour, nice appearance, good odour, good for taste, good for breakfast, perfect for snack, good for nutrition, and healthy. Participants were not rewarded for their participation.

### 2.8. Statistical Analysis

The experimental data, except the sensory analyses, were analysed by a one-way analysis of variance (ANOVA) using the Statistica v10.0 software (StatSoft, Inc., Tulsa, OK, USA). Fisher’s least significant differences (LSD) test was applied to assess the difference between each pair of means, with 95% confidence.

The sensory analysis data were collected by using the software Smart Sensory Box, an innovative device for sensory analysis and consumer test management (Smart Sensory Solutions S.r.l., Sassari, Italy), and analysed using STATISTICA 12 for Windows. Overall liking scores were analysed by ANOVA, considering the samples as a fixed source of variation and consumers as the random effect. After determining the frequency of use of each sensory attribute, Cochran’s Q test, which was followed by the McNemar (Bonferroni) multiple pairwise comparison test (*p* < 0.05), was applied to the CATA data to identify significant differences between samples for each of the terms included on the CATA questionnaire. Then, correspondence analysis (CA) was performed on the frequency table to obtain a bi-dimensional representation of the samples and the relationship between terms and samples. Penalty analysis, which was carried out on the consumers’ responses to identify the drop in overall liking linked to a deviation from the ideal product for each attribute chosen, was performed using XLSTAT for Windows (Version 2020.1.2, Addinsoft, Paris, France).

## 3. Results and Discussion

### 3.1. Flour and Biscuit Chemical-Physical and Nutritional Characteristics

Data on the proximate composition of flours and biscuits are reported in Table 1. The proximate compositions of QF and RF obtained agree with those reported in the literature [45,46,47]. The substitution of RF with QF increased both the moisture and aw values of the biscuits, although QF had a lower moisture content than RF. This result may be attributed to a potentially higher water absorbing index of QF with respect to RF, since QF has been reported to have a higher fibre content. The Q75 and Q100 biscuits registered an aw value close to 0.7, which could allow mould growth.

The data obtained show that the specific formulation used, which includes eggs but no added shortening, produces biscuits with significantly higher protein and ash values and a lower lipid content with respect to that reported by Demir and Kilinç, Brito et al. [19,24] for quinoa-based optimized biscuits, thus, the biscuits in this study had a more balanced nutritional profile. Moreover, the variation in ashes, lipids and proteins was less pronounced when compared to that reported by Demir and Kilinç, [19]. All the above-cited nutritional components increased linearly (*p* < 0.05) as RF was progressively substituted by QF, whereas TC decreased linearly. This result can probably be attributed to the differences in the flour compositions, as shown in Table 1, and depended on the relative substitution level.

### 3.2. Polyphenol Content and Antioxidant Activity of Flour and Biscuits

The results of the polyphenol fractions, TP, TF, and AA are reported in Table 2. The data on TP and TF agree with those in the literature [48]. Substitution of QF notably improved the polyphenol profile of the biscuits. In particular, TP and TF flavonoids increased significantly and in a linear manner (*p* < 0.05), according to the level of substitution and as much as four-fold compared with the Ctrl biscuits. SP increased more than IP. This nutritional improvement was mirrored by a similar significant increase in AA. The content of TP and TF was higher than that reported for quinoa-based biscuits by Demir and Kilinç, [19], but lower of that of quinoa-based biscuits made with raw or germinated flour [49]. There is no official recommended daily intake (RDI) for polyphenols, but values have been suggested that range from 1 to 1.2 g per day [50,51]. If we consider a biscuit serving size of 55 g, as recommended by the FDA [52], only 10% of the polyphenol RDI would be consumed from our biscuits made with 100% quinoa flour; thus, we can exclude potential problems related to an excessive intake of this compound.

### 3.3. Biscuit Colour and Texture

Biscuit colour and texture parameters are reported in Table 3. As expected, biscuit colour was affected by the rate of QF supplementation. The L and a* values progressively decreased and increased, respectively, following the rise in QF substitution. Lower L and higher a* values indicate that the QF samples were darker and redder than the Ctrl samples, as QF is browner than RF due to the fact that it is richer in polyphenols and ashes ( Table 1; Table 2), which directly affect the colour, and due to the higher protein content that promotes non-enzymatic browning via the Maillard reaction. The browning effect of QF has been previously reported for GF [24] and wheat-containing biscuits [19,23,49], and this may be considered a positive factor as one of the negative attributes of many GF bakery products is their lighter colour compared with conventional baked foods.

The ladyfinger biscuits produced in this study had a soft texture and are thus different to those described by Edoura-Gaena et al. [53], who reported a crunchy and crumbly texture. This is an important difference considering that the hardness values were significantly lower following QF substitution, with a progressive decrease in values as the rate increased. The lower hardness, or greater softness, of quinoa-based biscuits was confirmed by sensory analysis, as will be discussed in the following paragraph. Our data seem to disagree with the results obtained for other quinoa-based GF sweet bakery foods [19,23,24], but agree with those reported by Jan et al. [49]. These apparent differences may be due to the type of texture test used. Here, we applied the puncture test, and the hardness values corresponded to a yield point that is the maximum force encountered by the probe before crust penetration, thus, it does not account for the texture inside the biscuits, as also reported [19,23,24], but solely provides information on the resistance offered by the biscuit crust to penetration, as also shown by Jan et al. [49].

### 3.4. Sensory Analysis

Cochran’s Q test data are reported in Table 4. Most of the terms selected by consumers differed significantly between the different samples tested (*p* < 0.05).

The higher the proportion of QF in the sample, the higher the frequency at which the herbaceous and bitterness attributes were chosen. On the contrary, the biscuit with the lowest percentage of QF (Q25) showed values very close to the Ctrl biscuit, with a significant difference regarding the Ctrl sample only in relation to the good for nutrition attribute. The Q25 sample also showed a higher citation rate for the attributes nice colour, good for breakfast, and nice appearance when compared to the other three biscuits (Q50, Q75 and Q100).

**Figure 1:**
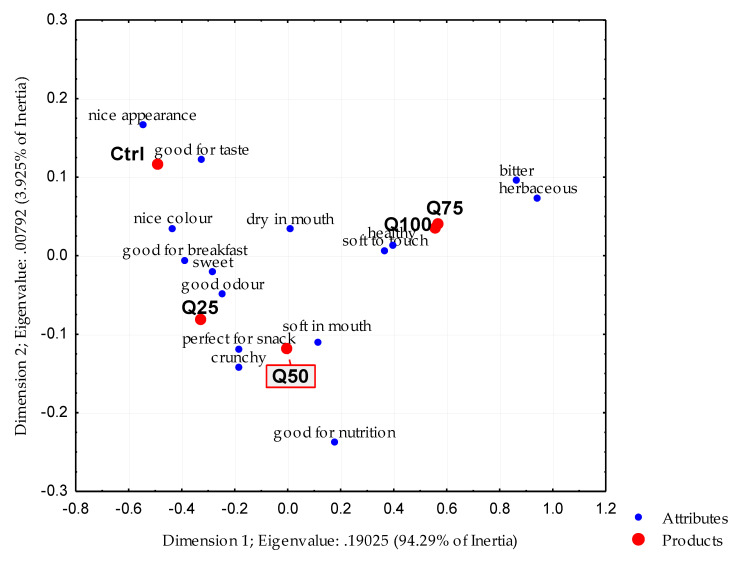
Correspondence analysis of ladyfinger biscuits made with RF only or at different QF substitution rates.

The CA results are reported in Figure 1. The first two dimensions explained 98.2% of total inertia. The Q75 and Q100 samples, which are very close in the bi-plot, were characterised by the attributes bitter and herbaceous, although considered by the consumers as healthier and softer to touch. The Ctrl sample, together with the Q25 and Q50 biscuits, correlated with all positive characteristics, including good odour, nice colour, nice appearance, sweet, good for taste, soft in the mouth, and so on.

**Figure 2:**
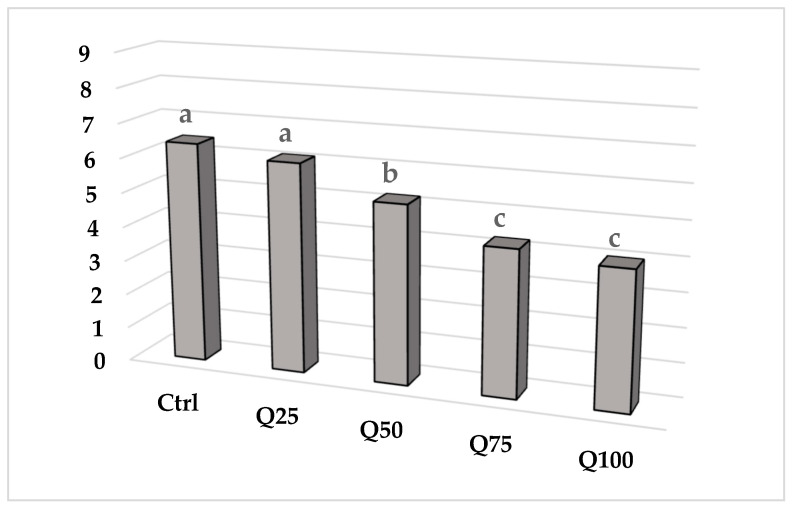
Influence of different substitution rates of QF on the ANOVA results of overall liking mean scores (*p* < 0.05) of GF ladyfinger biscuits. Values with the same letter do not differ significantly from each other according to LSD test (*p* < 0.05).

The ANOVA results regarding how consumers rated (or ‘liked’) the biscuits overall are reported in Figure 2. Consumers appreciated the Ctrl and Q25 samples the most, followed by the Q50, whereas the Q75 and Q100 biscuits were preferred the least.

The matrix correlation between the overall liking scores and the attributes showed a positive correlation between being liked and the attributes nice colour, sweet, good for breakfast, good for taste, nice appearance, and good odour. On the contrary, a negative correlation was found between the overall liking score and the attributes dry in mouth, herbaceous, and bitter.

**Figure 3:**
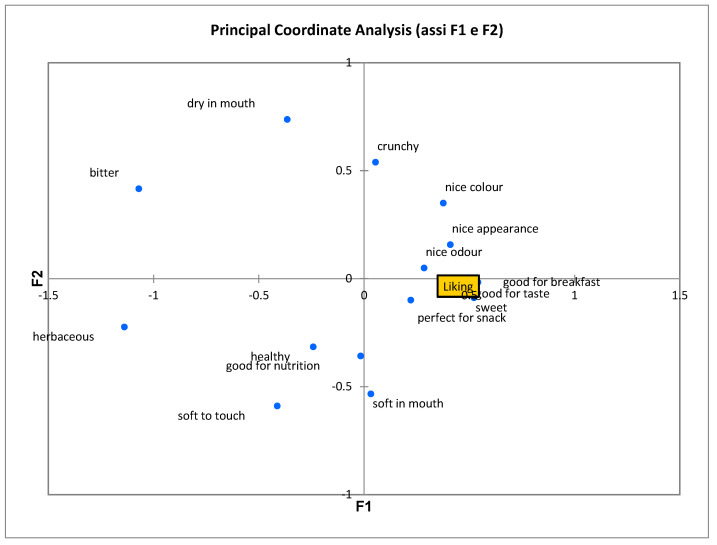
Principal coordinate analysis of ladyfinger biscuits made with RF only or at different QF substitution rates.

The results of principal coordinates analysis applied to the correlation coefficients are reported in Figure 3. About 43% of the variance is explained by the two dimensions. Again, we can observe that the overall liking score is associated with the attributes sweet, good for breakfast, good for taste, good odour, nice appearance, and nice colour.

Summary results of the penalty analysis put in evidence that herbaceous and dry in mouth are characteristics that the ideal biscuit must not have, whereas the main drivers of preference are the following attributes (Table 5): nice colour, sweet, good for breakfast, good for taste, nice appearance, soft in mouth, and good odour.

Unfortunately, despite the health benefits attributed to quinoa and recognised world-wide, the presence of bitter compounds in the quinoa flour, which include saponins and phenolic compounds, can affect the sensory characteristics of quinoa-based products [39]. The negative effects reported in this study confirm the data found in the literature [54,55], indicating that increasing the QF level in the biscuit formulation results in a decrease in colour, taste, odour, flavour, general appearance, and overall acceptability ratings of Q-based biscuits. New approaches aimed at decreasing the bitterness of quinoa flour need to be explored.

### 3.5. Volatiles

Following the analysis of the full scan GC/MS chromatogram, 20 compounds were tentatively identified in the headspace of the samples (Table 6). Specifically, the headspace of the analysed samples contained eight aldehydes, five alcohols, two terpenes, two nitrogen-containing derivatives, and three other compounds.

In general, the substitution of RF with QF resulted in a statistically significant increase in the majority of the compounds of each class, except for nitrogen-containing derivatives. Regarding the aldehydes, five out of eight increased after QF substitution; in fact, octanal (strong and fruity) and nonanal (waxy, green, fatty) were significantly higher compared with Ctrl biscuits at all the substitution rates. These aldehydes are well-known lipid oxidation products [56], and the change is surely due to the different lipid content of QF with respect to RF, as shown in Table 1. Moreover, the literature reports a higher content of unsaturated fatty acids (oleic, linoleic and linolenic) in QF when compared to RF, which are responsible for lipid oxidation [57]. A further three compounds—2-methyl-butanal (fruity, almond), benzaldehyde (bitter almond), and benzene acetaldehyde (honey, floral)—constitute three Strecker aldehydes attributable to the Maillard reaction [58,59], and are present at higher amounts in QF due to the higher protein content of QF and quinoa-based biscuits. The same picture described above can be applied to alcohols and furans; the biscuits made at all rates of QF supplementation were richer in comparison to the Ctrl samples, in 1-hexanol (sweet alcohol) due to lipid oxidation, and in benzyl alcohol (fruity, balsamic), phenylethyl alcohol (rose-like), and 2-pentyyl furan (floral, fruit) as a result of the Maillard reaction. Thus, the substitution of QF resulted in an increase in volatile compounds that are nearly always characterised by positive olfactory attributes (as can be appreciated by the terms reported in brackets above). However, in this kind of biscuit, it seemed that increasing percentages of substitution did not improve the sensory characteristics of these products. These data are, to the best of our knowledge, the first regarding the volatile characterization of GF biscuits, but further studies are needed to better explore their role in the sensory characteristics of GF products.

## 4. Conclusions

Improvements of the nutritional and sensory parameters have become paramount in the development of new GF bakery foods. With this in mind, this study evaluated, for the first time, the effect of partial or total substitution of RF with QF on the chemical-physical, nutritional, and sensory qualities of ladyfinger biscuits. Proximate and nutritional analyses revealed an improvement in all the parameters studied; that is, a higher content of proteins, lipids, ashes, TP and TF, as well as AA. The data show that the supplementation of GF biscuits with QF results in a more balanced nutritional profile compared with other quinoa-based sweet bakery products reported in the literature, and that the increase in TP does not pose any concern regarding potential toxicity. The only concern may be due to the increase in aw values in QF biscuits, caused by the higher water absorbing index of QF. Colour and texture were both improved by increasing rates of QF supplementation, as the quinoa-based biscuits developed a browner and more pleasant colour and were softer than rice-based ones. The sensory results revealed that the maximum substitution rate of QF should not exceed 50%, as higher supplementation impaired consumer acceptability due to the presence of herbaceous and bitter tastes, even if the consumers also rated these samples as healthier and softer to touch. The higher protein and lipid contents undoubtedly affected the build-up of the aroma profile of QF biscuits, which showed significantly higher levels of compounds derived from non-enzymatic lipid oxidation and the Maillard reaction, characterized by positive olfactory notes.

The results also indicate the potential for quinoa flour to improve the nutritional quality of GF biscuits due to its superior chemical composition, provided that the substitution rate does not exceed 50% in order to avoid sensory impairment of the product.

## Figures and Tables

**Table 1:** Proximate chemical composition of quinoa flour, rice flour, gluten-free control, and fortified ladyfinger biscuits (g/100 g d.m.).

Biscuits ^a^	Moisture			Aw			Proteins ^b^			Lipids			Ashes			TC ^c^		
Control	7.86	±	0.04e	0.556	±	0.01e	13.93	±	0.03e	5.6	±	0.2d	0.87	±	0.02e	71.84	±	0.08a
Q25	9.09	±	0.04c	0.615	±	0.01c	14.25	±	0.1d	5.96	±	0.02c	0.95	±	0.01d	69.84	±	0.13b
Q50	8.47	±	0.03d	0.585	±	0.01d	14.50	±	0.00c	6.12	±	0.1bc	1.07	±	0.01c	69.74	±	0.15b
Q75	10.46	±	0.01b	0.684	±	0.01b	15.95	±	0.03b	6.19	±	0.06ab	1.26	±	0.01b	66.13	±	0.04c
Q100	11.76	±	0.02a	0.704	±	0.01a	17.68	±	0.01a	6.35	±	0.05a	1.34	±	0.02a	62.83	±	0.03d
**Flours**																		
QF	12.37	±	0.05		-		17.61	±	0.01	4.06	±	0.04	2.51	±	0.01	63.45	±	0.05
RF	14	±	0.02		-		6.76	±	0.02	1.3	±	0.03	0.8	±	0.05	77.09	±	0.04

^a^ Mean Values ± standard deviation. Within columns, values (*n* = 3) with the same letter do not differ significantly from each other according to LSD test (*p* < 0.05). ^b^ Conversion factor from N to protein = 5.95. ^c^ TC: total carbohydrates calculated by indirect determination: TC = 100 − (moisture + lipids + protein + ash), quinoa flour (QF), rice flour (RF).

**Table 2:** Polyphenol fractions, total flavonoids, and antioxidant activity of quinoa flour, rice flour, gluten-free control, and fortified ladyfinger biscuits.

Biscuits ^a^	Polyphenol Fractions (mg GAE/100 g d.m.)									Flavonoids (mg CE/100 g d.m.)			Antioxidant Activity (%) *		
	Soluble			Insoluble ^b^			Total								
**Control**	**24.4**	**±**	**0.1e**	**16.3**	**±**	**0.5d**	**40.4**	**±**	**0.9e**	**5.1**	**±**	**0.5d**	**19.5**	**±**	**1.3d**
Q25	51.6	±	1.1d	28.2	±	1.1c	79.8	±	3.8d	13.3	±	0.1c	39.5	±	1.4c
Q50	79.8	±	4.0c	33.2	±	4b	113.0	±	7.3c	14.4	±	0.4c	66.6	±	2.2b
Q75	123.4	±	1.1b	35.8	±	1.1b	159.2	±	0.6b	16.7	±	0.0b	84.3	±	4.0a
Q100	142.8	±	1.2a	42.3	±	1.2a	185.1	±	6.2a	21.4	±	1.4a	84.3	±	0.5a
**Flours ^a^**															
QF	411	±	2.1	52.3	±	0.2	464.0	±	10.0	39.1	±	2.2	53.5	±	0.1
RF	23.0	±	1.1	17.4	±	0.5	40.0	±	4.6	0.9	±	0.5	26.3	±	0.2

^a^ Mean values ± standard deviation. Within columns, values (*n* = 3) with the same letter do not differ significantly from each other according to LSD test (*p* < 0.05). ^b^ Insoluble polyphenol fraction calculated by difference: Insoluble = (total − soluble). * Corresponding to 12 mg of quinoa and rice flour (36 mg of savoiardi biscuits), which consumed these percentages when 0.17 μmol of DPPH are available to react. GAE: gallic acid equivalent; CE: catechin equivalent.

**Table 3:** Influence of different substitution rates of QF on the crust colour and texture parameters of gluten-free (GF) ladyfinger biscuits.

Biscuits ^a^	Crust Colour									Texture								
	L			a*			b*			Hardness (N)			Slope (N/s)			Area		
**Control**	**76.25**	**±**	**1.06a**	**3.34**	**±**	**0.44a**	**25.26**	**±**	**0.57a**	**0.55**	**±**	**0.22a**	**0.61**	**±**	**0.29a**	**8.05**	**±**	**1.04a**
Q25	73.35	±	1.21b	4.05	±	0.90a	25.29	±	1.01a	0.51	±	0.10ab	1	±	0.55a	6.9	±	0.56a
Q50	68.84c	±	0.97c	5.45	±	0.13b	26.01	±	0.12a	0.4	±	0.15abc	0.53	±	0.21a	7.04	±	1.03a
Q75	66.73	±	0.93d	5.93	±	0.13b	26.67	±	0.11a	0.33	±	0.04bc	0.55	±	0.20a	9.55	±	3.62a
Q100	66.95	±	0.92d	4.29	±	0.72a	25.14	±	0.69a	0.2	±	0.11c	0.4	±	0.18a	8.04	±	2.24a

**^a^** Within columns, values (*n* = 3) with the same letter do not differ significantly from each other according to LSD test (*p* < 0.05).

**Table 4:** Influence of different substitution rates of QF on Cochran’s Q test results for GF ladyfinger biscuits.

CATA Attributes *	Biscuit Samples					
	*p*-values	Control	Q25	Q50	Q75	Q100
**nice colour**	**0.000**	0.718c **	0.592c	0.359b	0.155a	0.155a
**soft to touch**	**0.000**	0.282a	0.320ab	0.379ab	0.505b	0.495b
**sweet**	**0.000**	0.699c	0.563bc	0.495b	0.243a	0.214a
perfect for snack	0.053	0.146a	0.175a	0.126a	0.078a	0.068a
crunchy	0.054	0.155a	0.165a	0.155a	0.058a	0.087a
**good for nutrition**	**0.033**	0.078a	0.194b	0.165ab	0.126ab	0.155ab
**dry in mouth**	**0.011**	0.534b	0.534b	0.369a	0.379a	0.417a
**good for breakfast**	**0.000**	0.602c	0.553c	0.330b	0.155a	0.155a
**healthy**	**0.044**	0.107a	0.136a	0.146a	0.223a	0.204a
**good for taste**	**0.000**	0.417c	0.301bc	0.184ab	0.097a	0.155ab
**nice appearance**	**0.000**	0.631d	0.427c	0.214b	0.097ab	0.087a
**herbaceous**	**0.000**	0.019a	0.087a	0.340b	0.709c	0.786c
**soft in mouth**	0.099	0.427a	0.583a	0.544a	0.466a	0.476a
**good odour**	**0.000**	0.466c	0.456c	0.330bc	0.126a	0.243ab
**bitter**	**0.000**	0.029a	0.039a	0.126ab	0.252bc	0.291c

* In bold are the significant attributes following the Cochran’s Q test (*p* ≤ 0.05). ** Frequency relative values within rows, values with the same letter do not differ significantly from each other according to the McNemar (Bonferroni) multiple pair-wise comparison test (*p* < 0.05). CATA (check-all-that-apply).

**Table 5:** Summary table of the penalty analysis results.

Must Have	Nice to Have	Does Not Influence	Does Not Harm	Must Not Have
nice colour		soft to touch	crunchy	dry in mouth
sweet			good for nutrition	herbaceous
good for breakfast			healthy	
good for taste				
nice appearance				
soft in mouth				
good odour				

**Table 6:** Volatile organic compounds tentatively identified in ladyfinger GF biscuits by HS-SPME-GC/MS. Results are expressed as chromatogram peak area [×10^6^].

Volatile Compounds	Samples					RI
	Control	Q25	Q50	Q75	Q100	
*Aldehydes*						
2-methyl-butanal	1.13d *	2.10b	1.22c	3.51a	1.95b	919
Pentanal	0.23a	0.22a	0.16b	0.21a	0.26a	986
Hexanal	2.57a	2.54a	2.42a	2.04b	1.94b	1086
Heptanal	1.85b	2.55a	1.72b	2.16b	1.80b	1186
Octanal	2.26c	4.30a	2.97b	3.71a	3.85b	1290
Nonanal	8.13b	13.83a	12.45a	12.03a	9.10b	1399
Benzaldehyde	4.68b	4.53b	8.48b	13.71a	11.73a	1555
Benzene acetaldehyde	1.41c	1.33c	1.64c	3.03a	2.50b	1673
*Alcohols*						
1-hexanol	1.04c	2.13b	4.61a	4.19a	4.56a	1349
1-octen-3-ol	2.65c	2.70a	1.64b	1.80b	1.64b	1439
1-ethynyl-cyclohexanol	0.79b	0.67a	0.81a	0.68a	0.82a	1643
Benzyl alcohol	Nde	0.53d	0.89c	1.04b	1.36a	1841
Phenylethyl alcohol	0.43e	0.54d	0.78c	1.05b	1.19a	1866
*Terpenes*						
α-Pinene	0.27d	0.81c	1.81b	3.03a	3.11a	1028
D-Limonene	1.01d	1.00d	1.83c	2.19b	3.70a	1190
*Nitrogen-containing derivatives*						
Methyl-pyrazine	1.06a	1.35a	0.90a	1.68a	2.37a	1286
Pyrazine, 2,3-dimethyl-	13.81a	15.70a	7.16b	15.75a	7.27b	1341
*Others*						
Octane	0.46c	0.61b	0.61b	0.79a	0.66ab	800
Toluene	0.45b	0.60a	0.57a	0.53a	0.45b	1051
2-pentyl-furan	2.76d	3.63c	3.78c	5.94b	6.61a	1226

RI: linear retention indexes obtained on a 60 m VF-WAX capillary column. * Means within row, values (*n =* 3) with the same letter do not differ significantly from each other according to the LSD test (*p* < 0.05).
